# Diaphragmatic Stimulation: A case of Atrial Lead Dislodgement and Right Atrium perforation

**Published:** 2008-04-01

**Authors:** MH Namazi, R Karbasi-Afshar, M Safi, AR Serati

**Affiliations:** Cardiology research center, Modarres hospital, Shahid Beheshti university of Medical scinces, Tehran, Iran

**Keywords:** diaphragmatic stimulation, atrial pacing lead dislodgement, right atrium perforation

## Abstract

We report a 48 year old  male  who  presented  with  diaphragmatic stimulation. The biventricular implantable cardioverter and defibrillator (CRT-D) was implanted two weeks before admission and active fixation lead caused perforation of the right atrial wall. Echocardiography did not demonstrate pericardial effusion but Chest X-ray and computed tomography (CT) visualized the atrial screw helix outside the right atrial wall, penetrating through the right lung middle lobe. There was no atrial capture. After changing the pace mode DDDR to VVIR, diaphragmatic stimulation was disappeared. The atrial lead was repositioned and fixed again. During the hospital admission and after that the patient was well and free of any symptoms.

## Introduction

Diaphragmatic stimulation may be apparent and rarely requires fluoroscopy for confirmation. It may indicate direct stimulation of the diaphragm (left - sided) through a thin ventricular wall or less commonly, through a perforated ventricle. In the former case, reduction of output may relieve the problem. Another etiology for diaphragmatic stimulation (right - sided) is phrenic nerve stimulation with a displaced atrial or ventricular lead. Depending on the lead which is responsible, the corrective approach may entail inactivation of the channel, reduction of output, or repositioning of displaced lead.

Left diaphragmatic stimulation by pacemaker stimuli may occur during traditional pacing with or without lead perforation of the RV. Perforation must always be excluded when diaphragmatic pacing is observed. Late appearance of diaphragmatic pacing suggests insulation defects of the pacing lead. Left ventricular pacing from a coronary vein (in the absence of perforation) is an important and troublesome cause of diaphragmatic pacing during biventricular pacing for the treatment of heart failure. Contraction of the right diaphragm is related to a malpositioned right atrial electrode.

Here we report a patient with diaphragmatic stimulation due to atrial wall perforation who underwent re-operation and lead repositioning.

A 48 year old man with history of dilated cardiomyopathy and left bundle branch block who was eligible for CRT-D insertion underwent CRT-D implantation. During implantation due to inaccessible coronary sinus (CS) the septal lead was implanted. The procedure was performed under local anesthesia. The device (Epic  HF V-350) was implanted in the right pectoral area. The right ventricular (RV) bipolar lead (Riata 1571-65 Cm) was inserted via the right subclavian vein and positioned in the RV apex. The right atrial bipolar screw-in lead (Tendril SDX 1688T- 52 cm) was inserted via puncture of the right subclavian vein and positioned in the lateral side of right atrium. Then due to inaccessible coronary sinus (CS) instead of CS lead, RV bipolar screw- in lead (Medtronic 4076-58 Cm) was implanted. There was no puncture or attempt to puncture the left subclavian or jugular veins either before or during the implantation procedure. Atrial and ventricular sensing (4 and 16mv [RV] 10 mv [septal]) and pacing thresholds (0.5 and 0.25mv [RV] 1mv [septal]) were satisfactory. Lead impedance measurements were 600, 580 and 730 respectively. There was no diaphragmatic stimulation either with atrial or ventricular pacing at high output.

After procedure chest X- ray confirmed the proper positioning of the leads and also echocardiography for cardiac resynchronization therapy (CRT) optimization was acceptable. The patient did not have any pre-existing bronchopulmonary disease.

**Figure 1:**
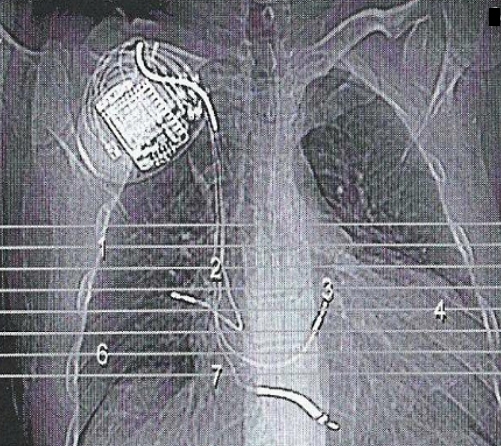


Two weeks after the hospital discharge, the patient was admitted because of diaphragmatic stimulation. CXR showed abnormal lead placement. Interrogation of the ICD showed 60 ppm spikes without any atrial capture but satisfactory atrial sensing. For relieving the patients symptoms we changed the mode from DDDR to VVIR and then stimulation terminated. After few days mild hemoptysis began and gradually increased but there was no sign of pericardial effusion in echocardiography. Chest CT scan (Figure 1) confirmed the atrial lead dislodgement which perforated the right atrial wall and reached the right middle lobe so alveolar hemorrhage occurred without any pericardial effusion.

The patient underwent repositioning in a safe setting with emergency surgery back up. Atrial lead was repositioned with guidance of transesophagial echocadiography and fixed at the other part of atrium. After the procedure, echocardiography showed mild pericardial effusion. After 3 months the patient showed no related symptoms.

## Discussion

Complications have been reported in up to 9% of atrial lead placements [1]. They are most often related to obtaining venous access (hemorrhage, pneumothorax: 2%) lead dislodgement (4.2%), inadequate pacing and sensing (3.5%) and acute pericarditis (5% in patients receiving active fixation atrial leads) [2,3]. Subclavian vein puncture may result in pneumothorax ipsilateral to the puncture. Myocardial perforation resulting in pericardial effusion or tamponade is rare and may require percutaneous drainage or open heart surgery. After reviewing literature we found a few reports of atrial leads perforating both pericardium and pleura, resulting in right - sided pneumothorax [4-7]. In all cases the pacing or ICD system was inserted in the left prepectoral area. Trigano et al. identified the possible risk factors for cardiac perforation using an active-fixation atrial lead [8]. Overscrewing of the lead, distal stylet insertion, abrupt lead withdrawal with extended screw or inadvertent displacement of the atrial lead during ventricular lead positioning were associated with more complications. The atrial lead was not repositioned after initial placement. Atrial and ventricular sensing and pacing parameters as well as fluoroscopic positions were satisfactory.  It is of interest to note that in our patient, neither the sensing parameters nor echocardiographic data could definitely define lead perforation as the cause of his symptoms. In two of the four previously reported cases the diagnosis was made with a PA chest X-ray, showing the presence of the atrial lead or helix outside the cardiac silhouette, while, in a third, clear protrusion could be visualized in a left anterior oblique fluoroscopic view (indicating the usefulness of different radiographic angles in suspected cases). In our patient, however, chest X-ray and CT visualized the tip of the atrial screw-in electrode just outside the contours of the right atrial appendage and into the right upper pulmonary lobe (Figure 1). As there was no gross protrusion of the lead body outside the cardiac silhouette we conclude that the perforation must have been caused by the extendable screw of the atrial lead. Since there are a few reports on right-sided pneumothorax in the absence of clear protrusion radiographically, the sensitivity of CT in these circumstances is unclear. Our finding, however, points to the additional diagnostic information that can be provided by CT. How far similar images may be found in other patients with atrial active-fixation leads (i.e. its specificity) has not been studied.

The CT images also illustrate the anterolateral right atrial wall, because of its thin aspect and its proximity to the overlying right lung, which predispose it to atrial and pleural perforations. Implanting the lateral and anterolateral right atrial wall also has been reported to predispose patients to post-implant pericarditis. Moreover, there seems to be an association between pericarditis and the use of pre-shaped J atrial leads [2,3], although no prospective data are available on this topic. It can be speculated that implantation of a straight screw-in atrial lead and/or anteromedial fixation (i.e. between the right atrial appendage and septum) might decrease the risk of this complication.

In two of the four described cases symptoms were found within hours of device implantation, while in the others symptoms emerged to 3rd, 4th or at the end of 30th day. Since the majority of device implantation procedures are uneventful after second day, we believe that common practice of early discharge (the day after the implantation) remains justified.

In summary, perforation of right atrial wall and reaching the right middle lobe without pneumothorax and pericardial effusion is very rare but a potentially catastrophic complication. Extraction of the lead is not mandatory; lead revision with repositioning was feasible in all described cases and allows maintenance of dual chamber pacing and sensing. The procedure should preferably be carried out in collaboration with a cardiac surgeon and/or after prior placement of a pericardial drain, since repositioning of the lead could potentially result in rapidly increasing pericardial effusion and tamponade.

## References

[R1] Connolly SJ, Kerr CR, Gent M (2000). Effects of physiologic pacing versus ventricular pacing on the risk of stroke and death due to cardiovascular causes. Canadian Trial of Physiologic Pacing Investigators. N Engl J Med.

[R2] Greene TO, Portnow AS, Stephen Huang SK (1994). Acute pericarditis resulting from an endocardial active fixation screw-in atrial lead. Pacing Clin Electrophysiol.

[R3] Sivakumaran S, Irwin ME, Gulamhusein SS (2002). Postpacemaker implant pericarditis: incidence and outcomes with active-fixation leads. Pacing Clin Electrophysiol.

[R4] Irwin JM, Greer GS, Lowe JE (1987). Atrial lead perforation: a case report. Pacing Clin Electrophysiol.

[R5] Wan-Jing H, Chi-Tai K, Kuo-Hong L (1999). Right pneumothorax resulting from an endocardial screw-in atrial lead. Chest.

[R6] Tran NT, Zivin A, Mozafferian D (2001). Right atrial perforation secondary to implantable cardioverter defibrillator insertion. Can Respir J.

[R7] Oginosawa Y, Abe H, Nakashima Y (2002). Cyclic GMP as mediator and biological marker of atrial natriuretic factor. Pacing Clin Electrophysiol.

[R8] Trigano AJ, Taramasco V, Paganelli F (1996). Incidence of perforation and other mechanical complications during dual active fixation. Pacing Clin Electrophysiol.

